# Predictors of the Development of Mental Disorders in Hospitalized COVID-19 Patients without Previous Psychiatric History: A Single-Center Retrospective Study in South Korea

**DOI:** 10.3390/ijerph19031092

**Published:** 2022-01-19

**Authors:** Jangrae Kim, Yae Eun Seo, Ho Kyung Sung, Hye Yoon Park, Myung Hwa Han, So Hee Lee

**Affiliations:** 1 Department of Psychiatry, National Medical Center, Eulji-ro 245, Jung-gu, Seoul 04564, Korea; jrkim2@nmc.or.kr (J.K.); t0916s@nmc.or.kr (Y.E.S.); hayround@kihasa.re.kr (M.H.H.); 2 Institute for Public Healthcare, National Medical Center, Eulji-ro 245, Jung-gu, Seoul 04564, Korea; 3 National Emergency Medical Center, National Medical Center, Eulji-ro 245, Jung-gu, Seoul 04564, Korea; 4 Department of Psychiatry, Seoul National University Hospital, Daehak-ro 101, Jongno-gu, Seoul 03080, Korea; psychepark@gmail.com; 5 Department of Psychiatry, Seoul National University College of Medicine, Daehak-ro 103, Jongno-gu, Seoul 03080, Korea

**Keywords:** COVID-19, mental disorder, cohort study, prognosis, Republic of Korea

## Abstract

The objective of this study was to investigate the predictors for new-onset mental disorders among patients with mild to moderate COVID-19 illness during hospitalization. A retrospective cohort study was performed in patients with confirmed COVID-19 admitted to a nationally designated hospital between 1 February and 30 June 2020. Demographic, clinical, psychological assessments, and psychiatric outcomes were obtained from electronic medical record review. Multivariate logistic regression analysis was used to identify predictors of new-onset mental disorders. Among 185 patients, 130 had no history of mental disorders or cognitive impairment at the time of admission. Of 130 patients, 29 (22.3%) were newly diagnosed with mental disorders during hospitalization. The following factors were significantly associated with an increased risk of a psychiatric diagnosis: Charlson comorbidity index core ≥1 (adjusted odds ratio (aOR) = 5.115, 95% confidence interval (CI): 1.737–15.058), length of stay (aOR per 1-day increase = 1.067, 95% CI: 1.035–1.100), and self-reported depressive symptoms at the time of admission (aOR = 5.357, 95% CI: 1.745–16.444). The predictive accuracy of combining these risk factors was relatively high (area under curve = 0.851, 95% CI: 0.778–0.923). These potential risk factors could help to predict the new-onset mental disorder among hospitalized patients with COVID-19.

## 1. Introduction

By the end of 2021, 278,714,484 cases of COVID-19 had been reported worldwide [1]. As the coronavirus disease 2019 (COVID-19) has rapidly spread worldwide, mental health among patients with COVID-19 is an emerging and important public health issue [2,3,4].

There is a growing body of evidence that has identified a high incidence of developing psychological sequelae in patients with COVID-19. In a cohort study from China, 23% survivors of the diseases reported anxiety or depression during the 6 months follow-up period [5]. A study using Korean health insurance claim data showed that COVID-19 survivors had a greater risk of developing mental illness than the rest of the population [6]. Psychological morbidities, including post-traumatic stress disorder (PTSD), anxiety, and depression, were reported to be common in COVID-19 patients, along with immune function changes associated with self-reported depression during short-term follow-up after discharge from hospital [7]. Analysis of data from patients with severe COVID-19 symptoms revealed neurological problems, such as delirium, dysexecutive syndrome, hypoxic encephalopathy, and encephalitis [8]. According to the results of telephone-based interventions for psychological problems in hospital isolated patients with mild to moderate COVID-19, clinically meaningful psychological symptoms such as anxiety, depression, insomnia, and suicidal ideation were found [9]. Among mild or moderate COVID-19 patients, suicidal attempts or violent behaviors during isolated hospitalization have been reported and might be challenging problems [10,11]. Previous psychiatric history is one of the important risk factors for depression and anxiety among survivors of COVID-19. Perceived stigma and history of psychiatric treatment affected PTSD symptom severity as psychological consequences of survivors of COVID-19 pneumonia 1 month after discharge [12]. For depression and anxiety, previous psychiatric history and stigma of COVID-19 infection were significant risk factors among the patients with mild illness [13].

However, the new-onset mental disorders among COVID-19 patients without previous psychiatric history undergoing treatment have yet to be sufficiently investigated. Additionally, effectiveness about psychological tests on admission to predict developing mental disorders during acute phase of COVID-19 would be important, because recognizing the risk group who needed psychiatric attention among isolated patients with COVID-19 as early as possible would help to advance the quality of emotional care and to prevent self-endangering or violent behavioral problems due to mental health problems. Therefore, we aimed to determine the proportion of patients newly diagnosed with mental disorders among the patients with COVID-19 during the acute phase of COVID-19, and to identify the risk factors.

## 2. Methods

### 2.1. Study Design and Subjects

**Figure 1:**
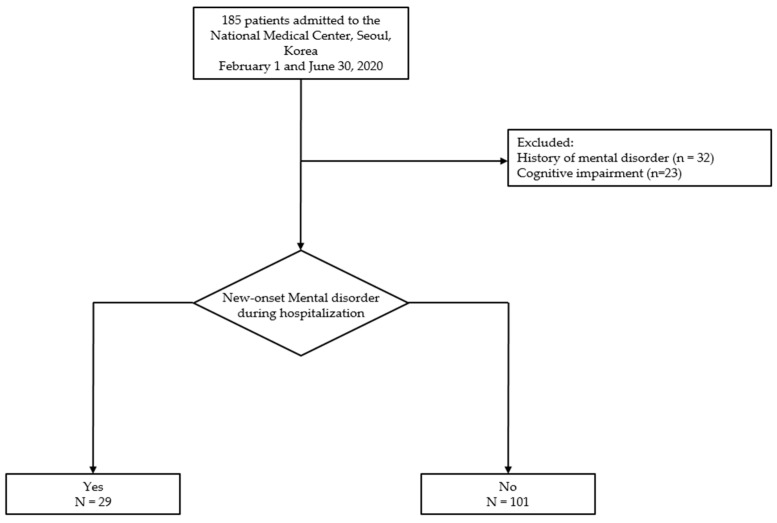
Flow chart of patients with COVID-19 admitted to the National Medical Center between 1 February and 30 June 2020.

This was a single-centered, retrospective cohort study. Inclusion criteria of this study were laboratory-confirmed COVID-19 patients admitted to the isolation wards of the National Medical Center (NMC), Seoul, South Korea between 1 February and 30 June 2020 and COVID-19 patients who could complete the psychological questionnaires when admitted in the hospital. Patients with a history of mental illness and those who were unable to the complete the psychological instruments due to diminished consciousness or substantial deterioration of the medical condition were excluded. Figure 1 shows the flow of study patients.

According to the guidelines of the Korea Disease Control and Prevention Agency (up to June 2020), confirmed cases were those with a positive real-time reverse transcription polymerase chain reaction (rRT-PCR) assay result for SARS-CoV-210. For discharge from hospital and cessation of isolation, negative rRT-PCR results were needed at 24 h intervals.

The NMC is a 480-bed public hospital in Seoul, Korea. The Korean government designated The NMC as a hospital for infectious diseases in 2015. After the pandemic reached Korea, the hospital’s resources have been concentrated on the management of COVID-19 patients; all wards that include negative pressure rooms have been dedicated to these patients. Based on experience of the MERS outbreak in 2015, all patients with COVID-19 admitted to the NMC were assigned to psychiatrists so that their mental health status could be monitored [14]. We offered three routes to access mental health services during hospitalization: psychological tests (self-report questionnaires) within a few days of admission; daily telephone calls to determine whether any mental issues were interfering with daily life; and face-to-face interviews on patient request or the emergence of acute psychiatric symptoms.

### 2.2. Outcome Variable

The outcome of interest was newly developed mental disorders among the patients during hospitalization. Mental disorders were diagnosed by two psychiatrists based on telephone or face-to-face interviews, along with thorough investigation of medical records and checking for signs. Patients’ family members or assigned nurses were contacted as needed for more information. The Diagnostic and Statistical Manual of Mental Disorders, 5th edition (DSM-5), was used for final diagnosis by the psychiatrists. Psychotropic medications were prescribed in accordance with the diagnosis, with consideration of the patients’ condition and progression.

### 2.3. Psychological Instruments

The psychological instruments used herein included the Patient Health Questionnaire-9 (PHQ-9), Generalized Anxiety Disorder-7 (GAD-7), Patient Health Questionnaire-15 (PHQ-15), PTSD Checklist for DSM-5 (PCL-5), and P4 Screener. We used the short versions of the Patient Health Questionnaire-2 (PHQ-2) and Korean version of the Primary Care PTSD Screen for DSM-5 (K-PC-PTSD-5) for patients aged 65 years or older.

We used the PHQ-9 to assess the severity of depression [15]. This questionnaire includes nine items based on the criteria for depression of the Diagnostic and Statistical Manual of Mental Disorders, 4th edition. Each item is scored from 0 to 3, for an overall severity score of 0–27. We considered depression to be present when the total score was ≥10. The Korean version of the PHQ-9 has been shown to be a reliable and valid tool for screening and assessment of depressed patients [16]. The Cronbach’s alpha coefficient was 0.85.

The PHQ-2 is a shorter instrument used to identify depression more rapidly [17]. The PHQ-2 has a validated Korean translation and has shown good validity (Cronbach’s alpha = 0.94); the optimal cut-off score for depression is 3.

The GAD-7 is a screening tool used to measure the severity of anxiety over the past 2 weeks. It consists of seven items rated on a four-point Likert-type scale ranging from 0 (Not at all) to 3 points (Nearly every day). A total score ≥ 10 is considered to be clinically significant. The Korean version of the GAD-7 showed excellent internal consistency (Cronbach’s alpha = 0.88) [18].

The K-PC-PTSD-5 is a brief instrument used to screen for PTSD symptoms according to the DSM-5 criteria. The K-PC-PTSD-5 has shown good internal consistency (Cronbach’s alpha = 0.87) [19]. We considered a score of 3 as the threshold for clinically significant PTSD symptoms.

The PCL-5 is a self-rating questionnaire to screen for PTSD symptoms based on the DSM-5 criteria. It includes 20 items, each scored from 0 to 4. A total score of 33 is the cut-off for PTSD diagnosis [20]. The Cronbach’s alpha was 0.94.

The P4 Screener was used to screen for suicidality based on past suicide attempts, suicidal ideation, probability of completing suicide, and preventive factors. Suicide risk is classified as minimal, low, or high. The P4 Screener is useful for assessing suicide risk in clinically depressed patients, including in the context of clinical research [21]. The low- and high-risk categories were taken to indicate suicidal status in this study.

### 2.4. Data Collection

Two experienced psychiatrists extracted data from electronic medical records using a standardized data collection form. Information concerning demographic characteristics, previous history of psychiatric symptoms, presence of pneumonia on admission, comorbidities, self-reported psychological assessments, oxygen therapy, psychiatric diagnoses, and psychotropic drugs prescribed during hospitalization (if any) was collected.

### 2.5. Statistical Analysis

We divided the patients into two groups according to whether or not they were newly diagnosed with any mental disorders during hospitalization. The demographic and COVID-19-related clinical characteristics and psychiatric information of both groups are presented as median and interquartile range (IQR) for continuous variables, and as frequency and percentage for categorical variables. Characteristics were compared between patient groups using the Mann–Whitney U test for continuous variables and the χ^2^ test or Fisher’s exact test for categorical variables, as appropriate.

Univariate logistic regression analysis was used to identify potential predictors of new-onset (during hospitalization) mental disorders. The potential predictors identified as significant in the univariate analysis were entered into multivariate logistic regression models, with the exclusion of highly correlated clinical factors using stepwise selection methods with entry and exit criteria of 0.05. The predictive performance of the multivariate logistic regression models was evaluated by area under the receiver operator characteristic curve (AUC) analysis. Given the potential for heterogeneity, we also performed a sensitivity analysis that excluded mental disorders associated with deterioration of the medical condition (i.e., delirium) from the analysis of the outcome variable.

A two-sided *p*-value < 0.05 was considered to indicate statistical significance for all test statistics. All analyses were performed using SAS version 9.4 (SAS Institute Inc.; Cary, NC, USA).

## 3. Results

### 3.1. Demographic and Clinical Characteristics

A total of 185 patients with COVID-19 were admitted to the isolation wards of the NMC between 1 February and 30 June 2020. Fifty-five patients were excluded due to a previous diagnosis of a mental disorder (*n* = 32) or the inability to complete the psychological instruments due to diminished consciousness or substantial deterioration of the medical condition (*n* = 23). Of the 130 eligible patients, 29 (22.3%) were newly diagnosed with mental disorders during their hospital stay.

Table 1 compares the demographic and clinical characteristics of patients with and without newly developed mental disorders during hospitalization. The median age was 58 (IQR: 35–68) years for patients with newly developed mental disorders and 40 (IQR: 27–58) years for patients without any newly developed mental disorders. Patients with newly developed mental disorders had a higher had a higher prevalence of Charlson comorbidity index (CCI) core ≥ 1 (44.8% vs. 17.8%, *p* = 0.003) including diabetes, prior myocardial infarction, mild liver disease, and acquired immune-deficiency syndrome [22]. Significant differences were also observed between the two groups in the incidence rates of pneumonia on admission (79.3% vs. 58.4, *p* = 0.040), oxygen therapy (31.0% vs. 7.9%, *p* = 0.003), and intensive care unit (ICU) admissions (17.2% vs. 5.0%, *p* = 0.029). Patients with newly developed mental disorders had longer median hospital stays (44 (IQR: 33–54) days vs. 26 (IQR: 16–39) days, *p* < 0.001) than those without newly developed mental disorders.

### 3.2. Psychological Assessment

Among the entire cohort, 23 (17.7%) patients reported depressive symptoms (score of ≥10 on the PHQ-9 or ≥3 on the PHQ-2), 7 (5.4%) reported significant PTSD symptoms (score of ≥3 on the PC-PTSD or ≥33 on the PCL-5), and 4 (3.1%) reported significant suicidal ideation; all of these patients completed the self-report questionnaires within a few days of admission (Table 2). Comparison between the two groups revealed that patients with new-onset mental disorders had a higher prevalence of depressive symptoms (37.9% vs. 11.9%, *p* = 0.001) than those without new-onset mental disorders. However, there was no significant difference between the two groups in PTSD symptoms and suicide ideation.

### 3.3. Newly Developed Mental Disorders and Prescribed Psychotropic Medications

Table 3 lists the newly developed mental disorders, which were clinically confirmed by two psychiatrists, and the prescribed psychotropic medications. Of the 29 patients with new-onset mental disorders, 14 (48.3%) had adjustment disorder, 10 (34.5%) had insomnia, 3 (10.3%) had panic disorder, and 2 (6.9%) had delirium. A total of 17 patients were prescribed psychotropic drugs; antianxiety medications (53.8%) were the most frequently prescribed, followed by antidepressants (23.1%) and antipsychotics (20.5%).

### 3.4. Predictors of New-Onset Mental Disorders

**Figure 2:**
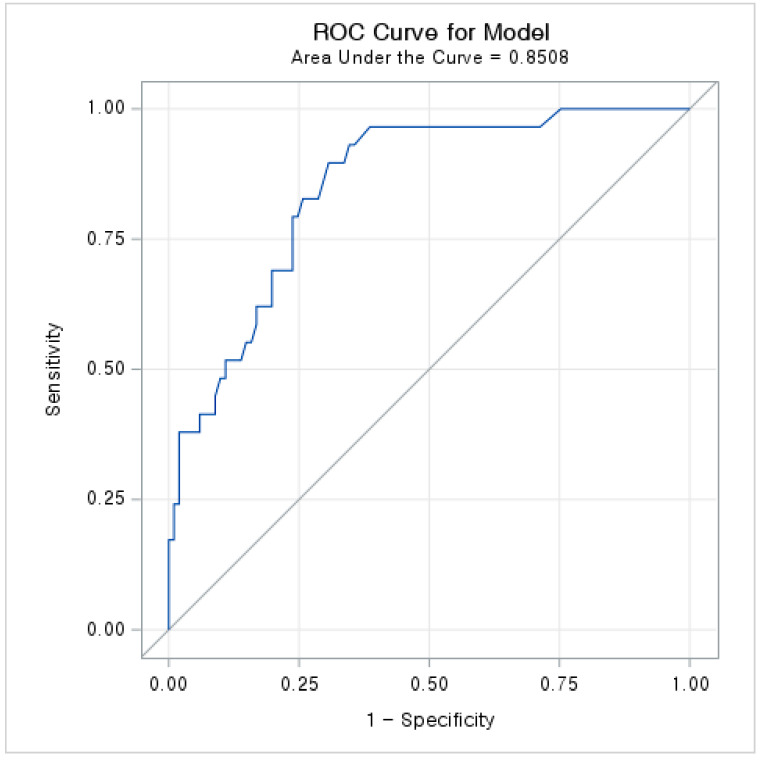
ROC curve and AUC assessing the predictive accuracy of multivariate model for new-onset mental disorder during hospitalization. ROC = receiver operating characteristic; AUC = area under curve.

In univariate analysis, age (unadjusted odds ratio (OR) per 1-year increase: 1.027, 95% confidence interval (CI): 1.004–1.051, *p* = 0.020), CCI score ≥ 1 (unadjusted OR: 3.747, 95% CI: 1.536–9.140, *p* = 0.004), duration of hospital stay (unadjusted OR per 1-day increase: 1.059, 95% CI: 1.030–1.089, *p* < 0.001), oxygen therapy (unadjusted OR: 5.231, 95% CI: 1.798–15.219, *p* = 0.002), ICU admission (unadjusted OR: 4.000, 95% CI: 1.071–1.941, *p* = 0.039), and self-reported depressive symptoms (unadjusted OR: 4.532, 95% CI: 1.732–11.864, *p* = 0.002) had *p*-values < 0.05. Among these variables, CCI score core ≥ 1, hospital length of stay, and self-reported depressive symptoms were included in the multivariate analysis using the stepwise selection method. In the multivariate analysis, CCI score core ≥ 1 (adjusted OR: 5.115, 95% CI: 1.737–15.058, *p* = 0.003), hospital length of stay (adjusted OR per 1-day increase: 1.067, 95% CI: 1.035–1.100, *p* < 0.001), and self-reported depressive symptoms (adjusted OR: 5.357, 95% CI: 1.745–16.444, *p* = 0.003) associated with a significantly increased risk of new-onset mental disorders (Table 4). Figure 2 presents the receiver operating characteristic (ROC) curve indicating the accuracy of the multivariate model for predicting new-onset mental disorders during hospitalization. The AUC was 0.851 (95% CI: 0.778–0.923).

In sensitivity analysis, we excluded two patients that developed delirium during hospitalization. The association was consistent in hospital length of stay (adjusted OR per 1-day increase: 1.059, 95% CI: 1.029–1.090, *p* < 0.001), and depression symptoms (adjusted OR: 5.830, 95% CI: 1.965–13.829, *p* = 0.001), except for CCI score core ≥ 1, which was associated with deterioration of medical condition and delirium (Table S1). The AUC of sensitivity analysis was 0.827 (95% CI: 0.740–0.915). The ROC assessing the predictive accuracy of sensitivity analysis is presented in Figure S1 (see Supplementary).

## 4. Discussion

In this study, 22.3% of patients with mild to moderate COVID-19 illness had newly diagnosed mental disorders during hospitalization. The most common newly diagnosed mental disorders were adjustment disorder and insomnia. This incidence and individual diagnosis are similar to the results of large cohort studies conducted in China and the US [5,23].

We also found 16.3% of the subjects had self-reported depressive symptom (PHQ-9 score ≥ 10 or PHQ-2 score 3) and 6.3% had self-reported PTSD symptom (PCL-5 score ≥ 33 or PC-PTSD score ≥ 3) on admission. A substantial portion of psychological problems on admission would be due to COVID-19 health anxiety [24]. The patients with COVID-19 might have excessive worry about their health condition or aggravation of the disease. In addition, COVID-19 patients might be distressed due to various troublesome events related with being quarantined, which included worries about taking care of children, leaving home, family members who may have also contracted COVID-19, or economic burdens suffered during hospitalization [25].

Our result suggested that several factors including comorbidity, hospital length of stay, and self-reported depressive symptom were associated with an increased risk of new-onset mental disorders. In particular, because early detection and rapid management are important, it is noteworthy that the usefulness of self-reported psychological tests was found. Our findings further suggest that psychiatric symptoms emerging during the initial phase of hospitalization predict the development of mental illness, highlighting the importance of involving mental health professionals in the earliest stages of hospitalization. Even though this result came from the context of a hospital in South Korea during the early pandemic period, early detection using self-reported psychological tests was demonstrated to be useful through Italian and US studies [26,27].

The hospital length of stay was strongly associated with the development of mental disorders and the risk was increased by approximately 7% per day. This is consistent with earlier studies on loneliness and depression in COVID-19 patients due to isolation during the initial phase of lockdown [28], where hospitalization duration was a major factor contributing to psychological distress [28,29]. As isolation to prevent the spread of SARS-CoV-2 could promote mental illness or exacerbate existing conditions [30,31,32], multidisciplinary programs with exercise and psychological supports are highly recommended for this population [9,33]. In addition, minimizing hospitalization for only isolation purposes could reduce the mental illness of patients with COVID-19.

Comorbidities showed a significant association with new-onset mental disorders. This is consistent with the result from a nationwide retrospective cohort study using Korean health insurance claims data [6]. This suggests that COVID-19 patients with underlying diseases need special psychiatric attention. It may be useful to detect high-risk groups by applying the brief screening instruments described above to patients with underlying diseases identified at the time of hospitalization and to perform psychiatric interventions at an early stage.

In the pandemic situation, it will be very important to consider the patient’s mental health needs [34]. The World Psychiatric Association position paper in 2020 insisted that the human rights of people with mental disorders must be protected, and appropriate and safe services provided for their treatment [35]. Investments in online mental health providing services, which build up the possibilities to reach all the mentally distressed people, were suggested to facilitate recovery from COVID-19 pandemic. Those strategies should be especially emphasized for the isolated people due to infection [36]. The strength of our study is that it showed the possibility of early detection of vulnerable people who can develop mental disorders.

## 5. Limitations

This study had several limitations. First, we included only patients admitted to a nationally designated COVID-19 hospital. Therefore, the results cannot be generalized to all patients with COVID-19. A larger and multi-centered study is needed to verify the results of this study. Second, the pandemic affected patients with chronic mental disorders, which is a very important public health issue, and we only included patients with no previous history of mental disorder. Third, self-reported psychiatric symptoms may be under- or over-reported. In particular, tests related to cognitive impairment were not performed. However, all of the psychological instruments used in this study were validated and all patients were assessed by a psychiatrist. Fourth, our study included a relatively small number of COVID-19 patients who progressed to severe illness. Therefore, the effect of severe illness, such as hypoxemia or medications, on the development of mental illness may not have been sufficiently evaluated. Finally, it is possible that the incidence of mental illness was underestimated because only the hospitalized period was evaluated.

## 6. Conclusions

This study provided evidence that a substantial proportion of COVID-19 patients without a history of psychiatric disorders may develop psychological symptoms and be diagnosed with a new-onset mental disorder. As well as comorbid physical conditions, depressive symptom should be checked for on admission using brief screening instruments. Furthermore, minimizing the length of hospital stay may reduce psychological suffering in COVID-19 patients. These potential risk factors could help to predict the new-onset mental disorder among patients with COVID-19 during hospitalization.

## Figures and Tables

**Table 1:** Baseline characteristics of the patients according to new-onset (during hospitalization) mental disorder status.

	Overall (*n* = 130)	Newly Developed Mental Disorders		*p* Value
		Yes (*n* = 29)	No (*n* = 101)	
Age, year	40 (28–61)	58 (35–68)	40 (27–58)	0.031
Sex				
Male	87 (66.9)	18 (62.1)	69 (68.3)	0.528
Female	43 (33.1)	11 (37.9)	32 (31.7)	
Marital status				
Single	56 (43.1)	8 (27.6)	48 (47.5)	0.056
Married	74 (56.9)	21 (72.4))	53 (52.5)	
Education level				
High school and under	65 (50.0)	19 (65.5)	46 (45.5)	0.058
University and higher	65 (50.0)	10 (34.5)	55 (54.5)	
Occupation				
Unemployed	58 (44.6)	13 (44.8)	45 (44.6)	0.979
Employed	72 (55.4)	16 (55.2)	56 (55.4)	
CCI score				
0	99 (76.2)	16 (55.2)	83 (82.2)	0.006 *
1–2	27 (20.8)	12 (41.4)	15 (14.9)	
3–4	0 (0)	0 (0)	0 (0)	
≥5	4 (3.1)	1 (3.5)	3 (3.0)	
Presence of pneumonia on admission				
No	48 (36.9)	6 (20.7)	42 (41.6)	0.040
Yes	82 (63.1)	23 (79.3)	59 (58.4)	
Oxygen therapy				
No	113 (86.9)	20 (69.0)	93 (92.1)	0.003 *
Yes	17 (13.1)	9 (31.0)	8 (7.9)	
Nasal cannula or simple facial mask	14 (10.8)	7 (24.1)	7 (6.9)	
HFNC or ventilator	3 (2.3)	2 (6.9)	1 (1.0)	
ICU admission				
No	120 (92.3)	24 (82.8)	96 (95.0)	0.029
Yes	10 (7.7)	5 (17.2)	5 (5.0)	
Hospital length of stay, days	31 (18–43)	44 (33–54)	26 (16–39)	<0.001
<28	59 (45.4)	5 (17.2)	54 (53.5)	0.001
≥28	71 (54.6)	24 (82.8)	47 (46.5)	

Values are expressed as *n* (%) or median (interquartile ranges), as appropriate. HFNC = high flow nasal cannula, ICU = intensive care unit, CCI = Charlson comorbidity index. * Fisher’s exact test.

**Table 2:** Baseline psychological assessment results of the patients.

	Overall *n* = 130	Newly Developed Mental Disorder		*p* Value
		Yes *n* = 29	No *n* = 101	
Depressive symptom				
PHQ-2 (*n* = 17)	1 (0–2)	1 (0–2)	1 (0–2)	0.733
PHQ-9 (*n* = 113)	3 (0–7)	8 (3–14)	3 (0–5)	0.002
Yes *	23 (17.7)	11 (37.9)	12 (11.9)	
No	107 (82.3)	18 (62.1)	89 (88.1)	0.001
PTSD symptom				
PC-PTSD (*n* = 17)	1 (0–2)	1 (0–1)	1 (1–3)	0.180
PCL-5 (*n* = 113)	3 (0–10)	7 (1–20)	2 (0–7)	0.047
Yes ^†^	7 (5.4)	0 (0.0)	7 (6.9)	
No	123 (94.6)	29 (100.0)	94 (93.1)	0.145
Suicide idea				
Yes	4 (3.1)	2 (6.9)	2 (2.0)	
No	126 (96.9)	27 (93.1)	99 (98.0)	0.177

Values are expressed as *n* (%) or median (interquartile ranges), as appropriate. * PHQ-9 score ≥ 10 or PHQ-2 score 3. ^†^ PCL-5 score ≥ 33 or PC-PTSD score ≥ 3.

**Table 3:** New-onset mental disorders and psychotropic medications prescribed during hospitalization.

	Number (%)
New-onset mental disorders	
Delirium	2 (6.9)
Panic disorder	3 (10.3)
Adjustment disorder	14 (48.3)
Insomnia	10 (34.5)
Psychotropic medications *	
Antipsychotics	7 (20.5)
Antidepressants	9 (23.1)
Antianxieties	20 (53.8)

* The number of prescriptions.

**Table 4:** Univariate and multivariate logistic analysis identifying predictors of new-onset (during hospitalization) mental disorders.

	Univariate Analysis		Multivariate Analysis	
	OR (95% CI)	*p* Value	OR (95% CI)	*p* Value
Age, years *	1.027 (1.004–1.051)	0.020		
Female sex (vs. male)	1.318 (0.558–-3.112)	0.529		
Low education level (vs. high)	2.272 (0.961–5.369)	0.062		
Unemployed (vs. employed)	1.011 (0.441–2.320)	0.9792		
CCI score ≥ 1 (vs. 0)	3.747 (1.536–9.140)	0.004	5.115 (1.737–15.058)	0.003
Pneumonia (vs. no)	2.729 (1.022–7.283)	0.045		
Hospital length of stay, days ^†^	1.059 (1.030–1.089)	<0.001	1.067 (1.035–1.100)	<0.001
Oxygen therapy (vs. no)	5.231 (1.798–15.219)	0.002		
ICU admission (vs. no)	4.000 (1.071–14.941)	0.039		
Depressive symptom ^‡^ (vs. no)	4.532 (1.732–11.864)	0.002	5.357 (1.745–16.444)	0.003
PTSD symptom ^‡^ (vs. no)	<0.001	>999.999		
Suicide idea ^‡^ (vs. no)	3.667 (0.493–27.246)	0.204		

OR = odds ratio; CI = confidence interval; CCI = Charlson comorbidity index; ICU = intensive care unit; PTSD = post-traumatic stress disorder; * per 1 year increase; ^†^ per 1 day increase; ^‡^ reported on self-assessed psychological test on admission.

## Data Availability

The data are not publicly available due to data use restrictions contained in study participants’ information material.

## Supplementary Materials

The following are available online at https://www.mdpi.com/article/10.3390/ijerph19031092/s1, Table S1: Univariate and multivariate logistic analysis of predictors for newly developed mental disorder except delirium during hospitalization (sensitivity analysis), Figure S1: ROC curve and AUC of sensitivity analysis.
