# Supplementary material for: Predictors of the Development of Mental Disorders in Hospitalized COVID-19 Patients without Previous Psychiatric History: A Single-Center Retrospective Study in South Korea
Source: Int J Environ Res Public Health. 2022 Jan 19;19(3):1092. doi: 10.3390/ijerph19031092 (PMC8834137; doi:10.3390/ijerph19031092)
Supplement: Supplementary file 1 [file ijerph-19-01092-s001.zip › ijerph-1507319-supplementary.pdf]

Supplemental Material

Table S1. Univariate and multivariate logistic analysis of predictors for newly developed mental disorder except delirium during hospitalization (sensitivity analysis).

	Univariate Analysis		Multivariate Analysis	
	**OR (95% CI)**	***p* Value**	**OR (95% CI)**	***p* Value**
Age, years *	1.027 (1.004–1.051)	0.020		
Female sex (vs. male)	1.318 (0.558–3.112)	0.529		
Low education level (vs. high)	2.272 (0.961–5.369)	0.062		
Unemployed (vs. employed)	1.011 (0.441–2.320)	0.9792		
CCI score core ≥ 1 (vs. 0)	3.747 (1.536–9.140)	0.004	5.115 (1.737–15.058)	0.003
Pneumonia (vs. no)	2.729 (1.022–7.283)	0.045		
Hospital length of stay, days†	1.059 (1.030–1.089)	<0.001	1.067 (1.035–1.100)	<0.001
Oxygen therapy (vs. no)	5.231 (1.798–15.219)	0.002		
ICU admission (vs. no)	4.000 (1.071–14.941)	0.039		
Depressive symptom (vs. no)	4.532 (1.732–11.864)	0.002	5.357 (1.745–16.444)	0.003
PTSD symptom ‡ (vs. no)	<0.001	>999.999		
Suicide idea ‡ (vs. no)	3.667 (0.493–27.246)	0.204		

OR = odds ratio, CI = confidence interval, CCI = Charlson comorbidity index, ICU = intensive care unit, PTSD = post-traumatic stress disorder, * per 1-year increase, † per 1-day increase, ‡ reported on self-assessed psychological test at admission.

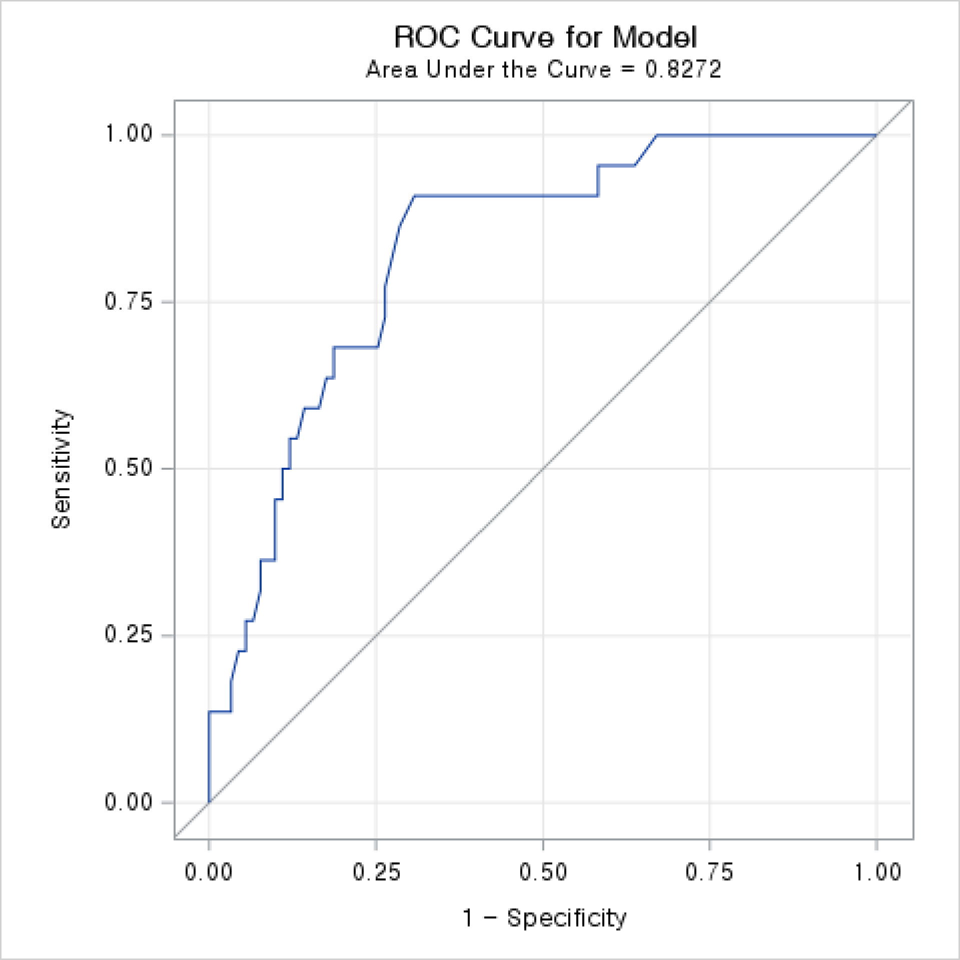

Figure S1. ROC curve and AUC of sensitivity analysis. ROC = receiver operating characteristic, AUC = area under curve.
